# Functional association of Loc1 and Puf6 with RNA helicase Dhh1 in translational regulation of *Saccharomyces cerevisiae* Ste12

**DOI:** 10.1371/journal.pone.0220137

**Published:** 2019-07-19

**Authors:** Daehee Jung, Jong Seok Seo, Jayoung Nam, Jinmi Kim

**Affiliations:** Department of Microbiology and Molecular Biology, College of Bioscience and Biotechnology, Chungnam National University, Daejeon, Republic of Korea; University of Parma, ITALY

## Abstract

Loc1 and Puf6, which are localized predominantly to the nucleus, are required for the localization and translational repression of the *ASH1* mRNA in the yeast, *Saccharomyces cerevisiae*. During its transport to the daughter cell, the *ASH1* mRNA is translationally repressed via associations with She2, Loc1, and Puf6. Here, we investigated the roles of Loc1 and Puf6 in the translation of mRNAs other than that encoding *ASH1*. In *loc1* or *puf6* deletion strains, expression of the mating-specific transcription factor, Ste12, was significantly increased at the post-transcriptional level. These phenotypes required the 5’ untranslated region (UTR) of *STE12*, which carries the putative Puf6-binding sequences. The RNA helicase, Dhh1, which is a known positive regulator for the translation of *STE12* mRNA, was found to be functionally connected with Loc1 and Puf6 in the context of Ste12 expression. Our results collectively show that the phosphorylation of the N-terminal Thr16 residue of Dhh1 affects the protein interactions of Dhh1 with Loc1 or Puf6, and consequently regulates Ste12 expression.

## Introduction

mRNAs are transcribed in the nucleus and transported to the cytoplasm, where they direct protein synthesis. The key regulatory steps for the eukaryotic gene expression include the nuclear assembly of pre-messenger ribonucleoprotein particles (mRNPs), the proper localization of the mRNA, and the initiation of translation. In the budding yeast, *Saccharomyces cerevisiae*, the Ash1 protein has been reported to be asymmetrically localized in daughter cells to repress the transcription of HO endonuclease, which is crucial for the mating type switch [1–3]. The *ASH1* mRNA is transcribed in the mother cell and transported to the distal tip of the daughter cell, where the protein is translated. During this transport, the translation of the *ASH1* mRNA is repressed by its associations with RNA-binding proteins such as She2, Puf6, Loc1, and Khd1 [4–8].

Puf6 belongs to the pumilio/fem-3 domain family whose members are characterized by a conserved RNA-binding domain with eight PUM (pumilio) repeats of ~ 36 amino acids [9,10]. Puf6 represses translation of the *ASH1* mRNA by binding primarily to its 3’-UTR which contains the conserved UUGU elements [5]. Loc1 has been implicated in the assembly of nuclear mRNPs [11,12]. Both Puf6 and Loc1 are nuclear proteins that are enriched in the nucleolus. The *ASH1* mRNA is exported to the cytoplasm along with Puf6, whereas Loc1 is removed from the *ASH1* mRNA complex prior to or during nuclear export [11]. Deletion of *LOC1* or *PUF6* decreases the efficiency of *ASH1* mRNA localization and up-regulates the cytoplasmic translation of the *ASH1* mRNA [5,11].

The Ste12 protein is the primary transcriptional activator responsible for initiating the transcription of about 200 mating-specific genes in *S*. *cerevisiae* [13,14]. Upon α-factor stimulation, Ste12 dissociates from its inhibitors, Dig1 and Dig2, and binds to promoters containing pheromone-responsive elements (PREs). Additionally, through its binding with the transcription factor Tec1, Ste12 functions as a key transcriptional regulator during the filamentous response [15,16]. Transcription of the *STE12* gene itself is activated by α-factor through four PREs located in its promoter [17]. In addition, *STE12* expression is reportedly regulated at the translational level under both filamentous growth and mating conditions [18–21].

The Dhh1 protein, which is a member of the DEAD-box RNA helicase family, functions as a mRNA decapping activator in the mRNA decay pathway and is a major component of the cytoplasmic mRNA granules that are known as P-bodies (processing bodies) [22,23]. Dhh1 has been widely studied as a translational repressor, but accumulating evidence shows that it also participates in translational regulation as a positive and gene-specific activator [18,19,24]. The *dhh1* deletion mutation significantly decreased the Ste12 protein level without altering the *STE12* transcript level during both the mating process and hyphal growth. High-throughput analysis using both ribosome profiling and RNA-seq experiments in *dhh1* mutant cells revealed that a significant number of selected mRNAs are positively regulated by Dhh1 [24].

In the present study, we investigated the potential involvement of Loc1 and Puf6 in the translation of mRNAs other than the *ASH1* mRNA. We found that Loc1 and Puf6 appear to translationally repress the *STE12* mRNA. The *loc1* or *puf6* deletion mutations increased *STE12* expression at the post-transcriptional level. Genetic and co-immunoprecipitation analyses revealed that Loc1 and Puf6 are functionally connected with the RNA helicase, Dhh1, in regulating Ste12 expression. The N-terminal phosphorylation sites of Dhh1 were found to regulate the association of Dhh1 with Loc1 or Puf6.

## Results

### The translational repressors Loc1 and Puf6 are functionally connected to Dhh1 in regulating Ste12 protein expression

**Fig 1:**
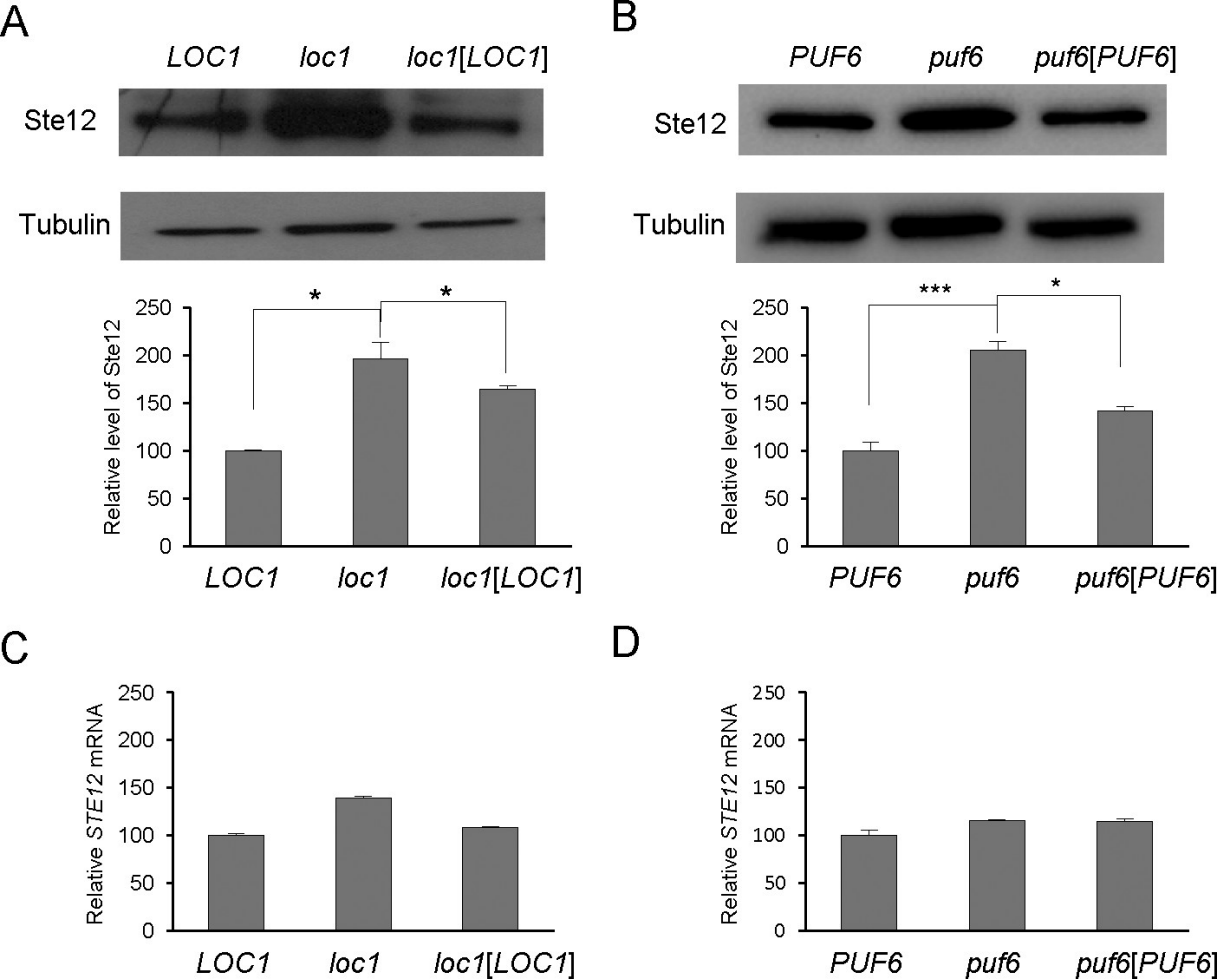
Ste12 protein levels were increased in *loc1* or *puf6* deletion strains. (A) Ste12-HA protein levels were measured by Western blot in wild-type cells, *loc1* cells, and *loc1* cells carrying the *LOC1* plasmid. Tubulin was detected as a loading control. A representative Western blot is shown. Graphs represent quantification of Ste12-HA to tubulin ratio (n = 3 independent replicates). Values are mean ± SD. *p<0.05 (B) Ste12-HA protein levels were assessed in wild-type cells, *puf6* cells, and, *puf6* cells carrying the *PUF6* plasmid. A representative Western blot is shown. Graphs represent quantification of Ste12-HA to tubulin ratio (n = 3 independent replicates). Values are mean ± SD. * p < 0.05. *** p < 0.005 (C) RNA prepared from the cultures listed in (A) was analyzed by quantitative RT-PCR. *STE12* mRNA expression was normalized against *ACT1* mRNA expression (error bars, mean + S.D.). (D) RNA prepared from the cultures listed in (B) was analyzed by quantitative RT-PCR. *STE12* mRNA expression was normalized against *ACT1* mRNA expression (error bars, mean + S.D.).

Loc1 and Puf6 are localized predominantly to the nucleus, and are required for the localization and translational repression of the *ASH1* mRNA [3,12,25]. We questioned whether Loc1 and/or Puf6 could translationally repress other mRNAs. Previous reports showed that the transcription factor, Ste12, is post-transcriptionally regulated under conditions that promote filamentous growth and mating [18–20]. Here, we analyzed the expression of Ste12 in *loc1* or *puf6* deletion strain (Fig 1A and 1B). Ste12-HA protein levels were found to be higher in *loc1* or *puf6* deletion mutant as compared with wild-type cells. Quantitation of *STE12* transcripts revealed that the *loc1* mutation caused a slight increase in the *STE12* mRNA level, and the *puf6* mutation did not significantly alter this level compared with the wild-type strain (Fig 1C and 1D). These results suggest that Loc1 and Puf6 repressed the expression of *STE12* at the post-transcriptional level.

**Fig 2:**
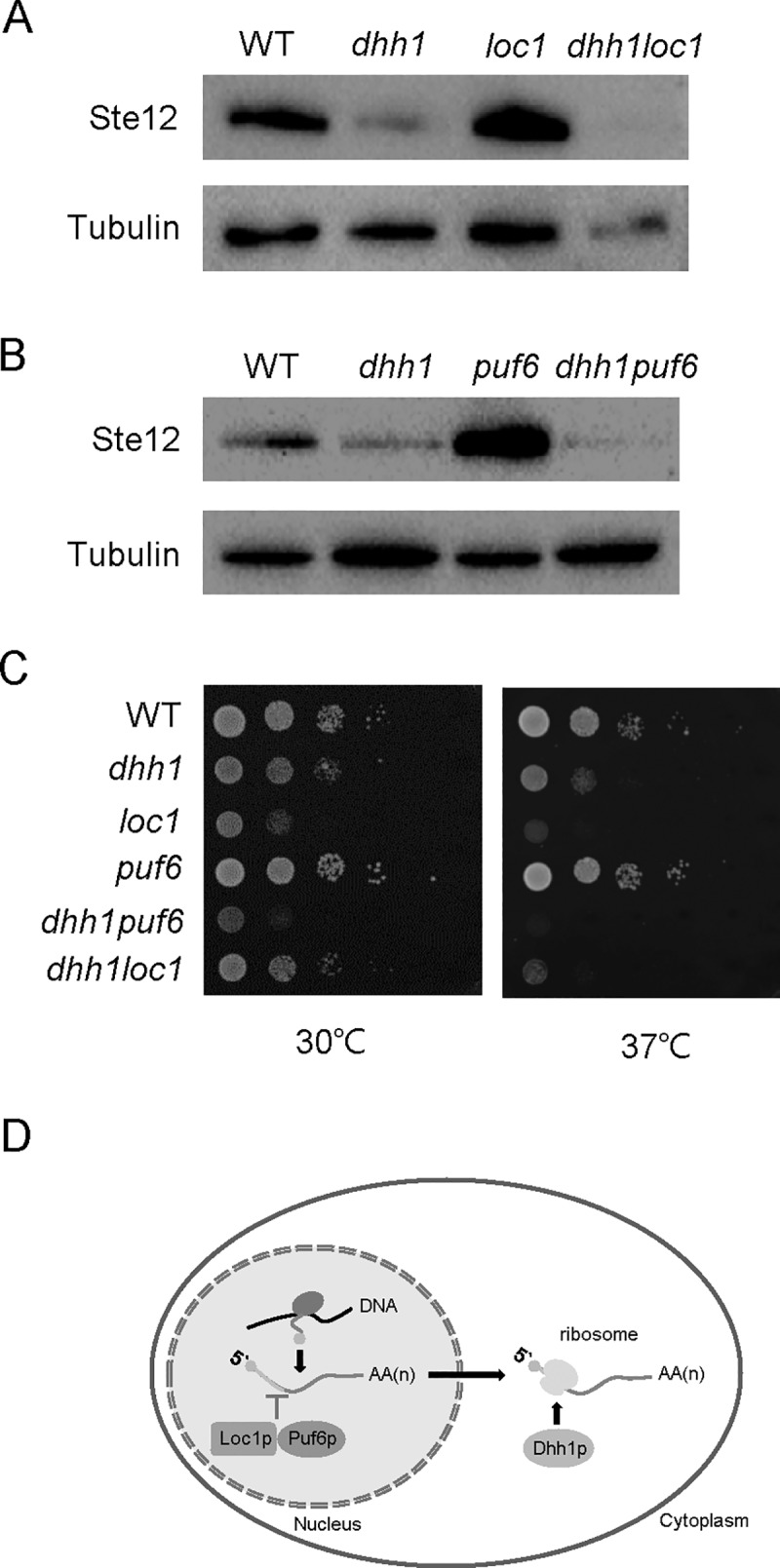
Genetic interactions of *dhh1*, *loc1*, and *puf6* mutations revealed by double-mutant analysis. (A) Ste12-HA protein levels were measured in the wild-type, *dhh1*, *loc1*, and *dhh1loc1* cells by Western blot analysis. Tubulin was used as a loading control. (B) Ste12-HA protein levels were measured in wild-type, *dhh1*, *puf6*, and *dhh1puf6* cells. (C) Growth defects of the *dhh1loc1* and *dhh1puf6* double-mutant strains. Ten-fold serial dilutions of overnight cultures of wild-type, *dhh1*, *loc1*, *puf6*, *dhh1loc1* and *dhh1puf6* cells were spotted on YEPD plates, which were incubated at 30°C and 37°C for 2 days and then photographed. (D) Model of the regulation of *STE12* mRNA by Loc1, Puf6, and Dhh1. Loc1 and Puf6 are localized predominantly in the nucleus and repress the expression of *STE12* at the post-transcriptional level. Dhh1 positively regulates the translation of the *STE12* mRNA, possibly downstream of Loc1 and Puf6.

We next examined the genetic interactions of Loc1 and Puf6 with the DEAD-box RNA helicase, Dhh1, because Ste12 protein expression is known to be decreased in *dhh1* deletion or domain-specific mutant strains [18–20]. The *STE12* transcript is not affected by *dhh1* mutations [18, 19]. We deleted *DHH1* from the *loc1* or *puf6* strains and examined Ste12 expression (Fig 2A and 2B). The *dhh1loc1* and *dhh1puf6* double deletion strains showed Ste12 levels similar to that of the *dhh1* strain, indicating that the *dhh1* deletion abolished the de-repressed state of Ste12 expression in *loc1* or *puf6* cells. We hypothesize that Dhh1 regulates Ste12 translation in functional association with Loc1 and Puf6, possibly downstream of these factors (Fig 2D).

We analyzed the growth phenotypes of the *dhh1loc1* and *dhh1puf6* double-deletion strains (Fig 2C). Previous reports indicated that the *dhh1* deletion mutant has a growth defect at 37°C and the *loc1* deletion mutant shows a severely slow growth phenotype, whereas deletion of *PUF6* does not cause any significant growth defect [12, 23, 26]. The *dhh1loc1* mutant strain showed a slow growth phenotype at 30°C and a growth defect at 37°C, and thus resembled the *dhh1* mutant. The *dhh1puf6* mutant strain showed a growth phenotype, almost similar to that of *dhh1* cells. These results suggest that Dhh1 interacts genetically with Loc1 and Puf6.

### Loc1- and Puf6-mediated repressions are *STE12* 5’-UTR-dependent

**Fig 3:**
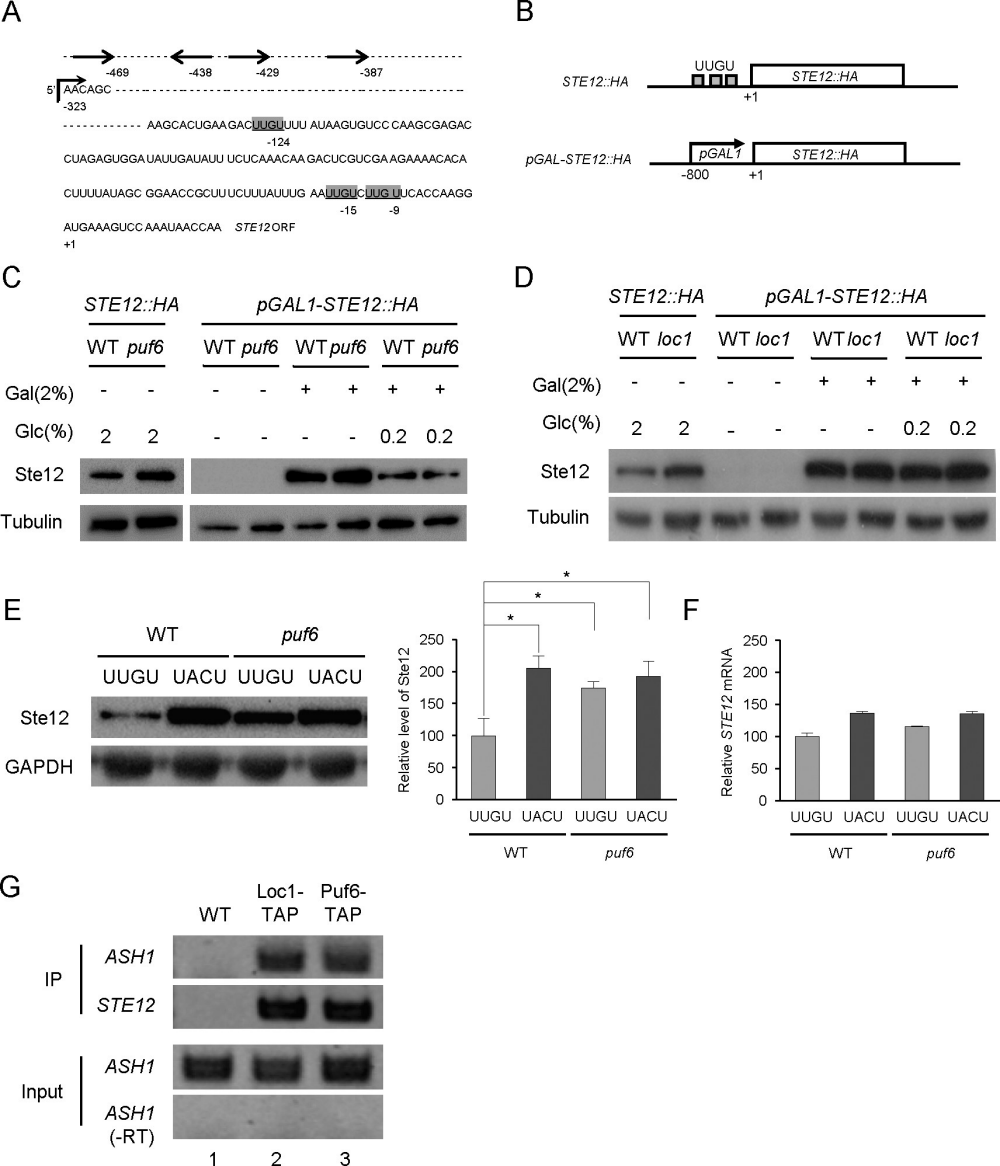
5’UTR-dependent repression of the *STE12* mRNA. (A) Schematic representation of the UUGU sequences in the 5’-UTR of *STE12* mRNA. The AUG start codon is indicated as +1 in the *STE12* ORF. UUGU sequences were found at the -124, -15, and -9 positions. The 5’-UTR starts at -323 position as indicated with a right angle arrow. Sequences indicated with arrows represent the pheromone responsive elements (PREs). (B) Diagrams showing the wild-type *STE12*::*HA* and *pGAL-STE12*::*HA* constructs. The p*GAL-STE12*::*HA* plasmid carried a 800-bp fragment containing the *GAL1* promoter region. (C) Western blot analysis of Ste12-HA proteins prepared from wild-type and *loc1* cells carrying the *STE12*::*HA* or p*GAL-STE12*::*HA* plasmids. For *STE12*::*HA* (lanes 1 and 2), cells grown to the early log phase (OD_600_ = 1.0) in 2% glucose medium were used as a control. For galactose induction, cells were grown in SC medium containing 2% raffinose to the early log phase (OD_600_ = 1.0), and induced with 2% galactose or 2% galactose plus 0.2% glucose for 2 hours. Lanes 3 and 4, 2% raffinose as a sole carbon source; lanes 5 and 6, 2% galactose was added for *GAL1* promoter induction; lanes 7 and 8, 2% galactose plus 0.2% glucose. Tubulin was detected as a loading control. (D) Western blot analysis of Ste12-HA proteins prepared from wild-type and *puf6* cells carrying *STE12*::*HA* or *pGAL-STE12*::*HA* plasmids. Cultures and protein analysis were performed essentially as described in (C). (E) Ste12-HA protein levels in cells carrying the wild-type UUGU-STE12-HA or mutant UACU-STE12-HA construct, as revealed by Western blot analysis. Graphs represent quantification of Ste12-HA to GAPDH ratio (n = 2 independent replicates). Values are mean ± SD. *p < 0.05. (F) RNA prepared from the cultures listed in (E) was analyzed by quantitative RT-PCR. *STE12* mRNA expression was normalized against *ACT1* mRNA expression (error bars, mean + S.D.). (G) RNA immunoprecipitation of Loc1-TAP and Puf6-TAP from the cell extracts of the wild-type or TAP-tagged strain, followed by RNA purification and RT-PCR. *STE12-*specific primers were used for PCR. *ASH1* mRNAs were detected as a binding control.–RT indicates RT-PCR without reverse transcriptase.

Puf6 represses the *ASH1* mRNA by binding to its 3’-UTR, which carries multiple copies of the conserved UUGU element [5,7]. Our sequence analysis of the *STE12* mRNA identified UUGU sequences located at nine, 15, and 124 nucleotides upstream of the *STE12* start codon (Fig 3A). The length of *STE12* 5’-UTR has been reported to be 323 nucleotides [27, 28]. Three UUGU sequences were in this 5’-UTR. Four PREs (indicated as -387, 429, -438, and -469) were located in the *STE12* promoter region [17].

To assess whether the abilities of Puf6 and Loc1 to regulate Ste12 protein expression are mediated by the *STE12* 5’-UTR, we employed a p*GAL1-STE12* construct in which the promoter and upstream sequences of the *STE12* ORF were replaced with a heterologous *GAL1* promoter region (Fig 3B). The plasmid p*GAL1-STE12-HA* carries about 800 bp fragment of the yeast *GAL1* promoter inserted at position -1 of the *STE12* start codon. This construct contains the endogenous 3-’UTR of *STE12* mRNA. Expression of p*GAL1-STE12-HA* was induced with 2% galactose in wild-type, *puf6* mutant, and *loc1* mutant strains. In wild-type *STE12-HA* cells, the de-repression phenotypes caused by *puf6* or *loc1* mutation were evident in 2% glucose media (Fig 3C and 3D, lane 1, 2). The levels of p*GAL1-STE12-HA* expression were similar in wild-type and *puf6* mutant cells (lane 5, 6). To avoid artifacts caused by over-expression, the level of galactose induction was modulated by adding 0.2% of glucose to the media (lane 7, 8). We obtained similar results in the wild-type and *loc1* mutant cells (Fig 3D). These results suggest that Puf6 and Loc1 modulate *STE12* translation in a 5’-UTR-dependent manner. We further questioned whether the putative Puf-binding sites in the 5’UTR of the *STE12* mRNA are important for the translational repression of this transcript. The UUGU element at 124 nucleotides upstream of the start codon was mutated to UACU and the resulting plasmid, 5’UACU-STE12-HA, was introduced into the wild-type and *puf6* deletion strains. As shown in Fig 3E, the Ste12-HA protein level was significantly higher in a wild-type strain carrying the mutant 5’UACU-STE12-HA mutant compared to a wild-type strain carrying the wild-type 5’UUGU-STE12-HA (lanes 1 and 2). In a *puf6* deletion strain, de-repression of Ste12 expression was similarly observed for the wild-type 5’UUGU-STE12-HA and the mutant 5’UACU-STE12-HA (lanes 3 and 4). These results suggest that the UUGU elements in the 5’UTR of the *STE12* mRNA are critical for the regulation of *STE12* translation. The interaction between the *STE12* mRNA and the Loc1 or Puf6 protein in vivo was determined by RNA immunoprecipitation. In this assay, the Loc1-TAP or Puf6-TAP proteins were precipitated from the cell extracts and *STE12* mRNAs were detected by RT-PCR analysis. Both Loc1-TAP and Puf6-TAP interacted with the *STE12* mRNA, also with the *ASH1* mRNA (Fig 3G). It is highly likely that Loc1 and Puf6 bind to the *STE12* mRNA to regulate its translation.

### Dhh1 and Puf6 regulate the protein expression levels of Cln1 and Gpa2

**Fig 4:**
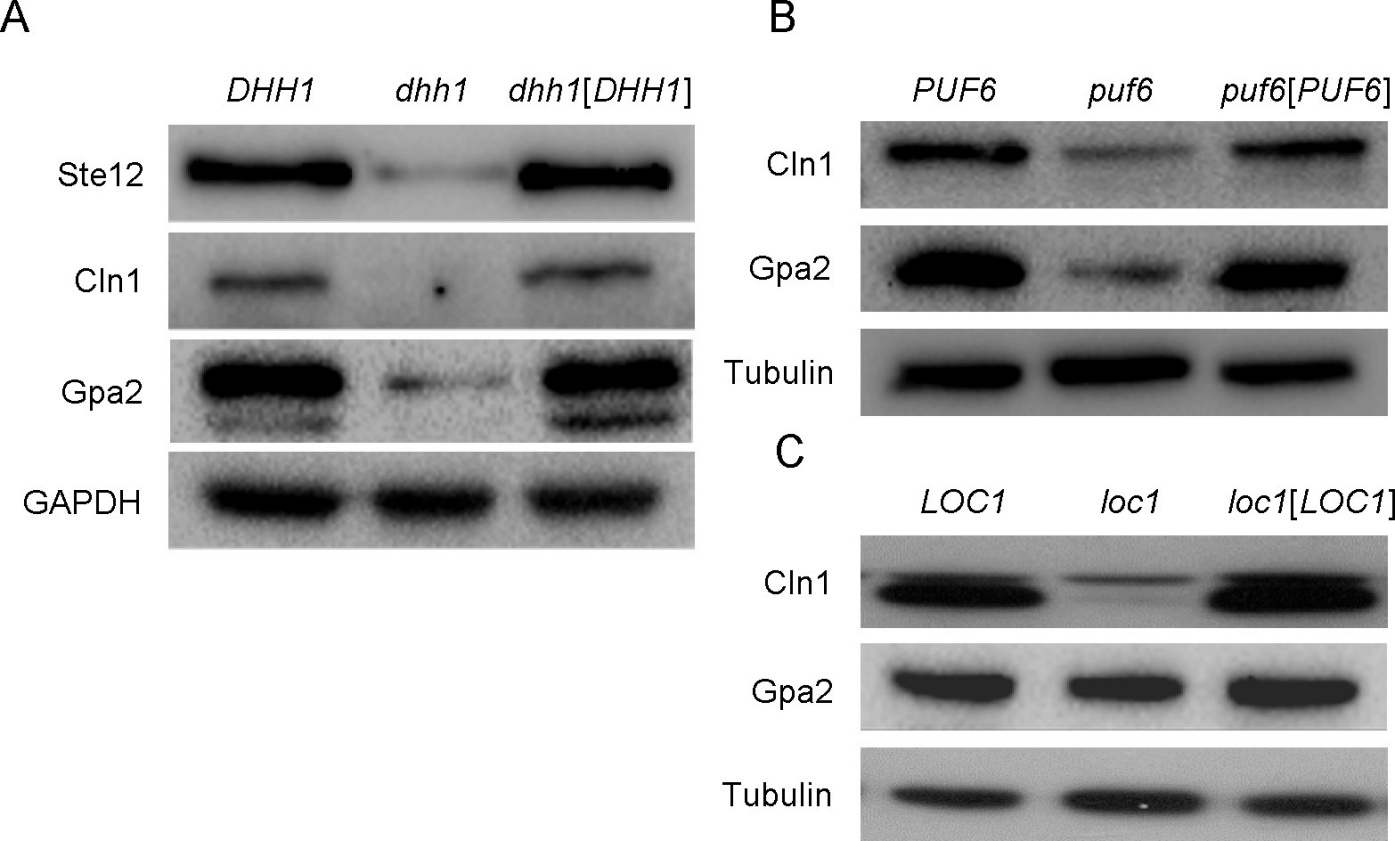
Effects of *dhh1*, *loc1*, and *puf6* mutations on the protein expression levels of Cln1 and Gpa2. (A) Western blots of Ste12-HA, Cln1-HA and Gpa2-HA proteins in the *dhh1* mutant strain. GAPDH was detected as a loading control. (B) Western blots of Cln1-HA and Gpa2-HA proteins in the *puf6* mutant strain. Tubulin was detected as a loading control. (C) Western blots of Cln1-HA and Gpa2-HA proteins in the *loc1* mutant strain. Tubulin was detected as a loading control.

In a screen performed using polyribosome fractionation, *STE12*, *GPA2*, *and CLN1* were identified as being translationally regulated during filamentous growth [19]. Cln1 is an mRNA target of 4E-BP-mediated translational repression during growth on rich media [26]. Here, we analyzed the protein expression levels of Cln1 and Gpa2 in *dhh1*, *loc1*, and *puf6* deletion strains. In the *dhh1* mutant strain, the protein levels of Gpa2 and Cln1 were decreased compared to those in wild-type cells, and thus paralleled Ste12 protein expression (Fig 4A). Interestingly, the protein levels of Gpa2 and Cln1 were decreased in the *puf6* and *loc1* mutant strains compared with wild-type cells, and thus contrasted with the Ste12 protein expression pattern (Fig 4B and 4C). The Gpa2 protein level was only slightly decreased in the *loc1* mutant strain. Together, these results suggest that *puf6* or *loc1* deletion had gene-specific effects on the expression levels of Ste12, Gpa2, and Cln1.

### Phosphorylation site mutations of *DHH1* affect Ste12 expression and the Puf6-Dhh1 interaction

**Fig 5:**
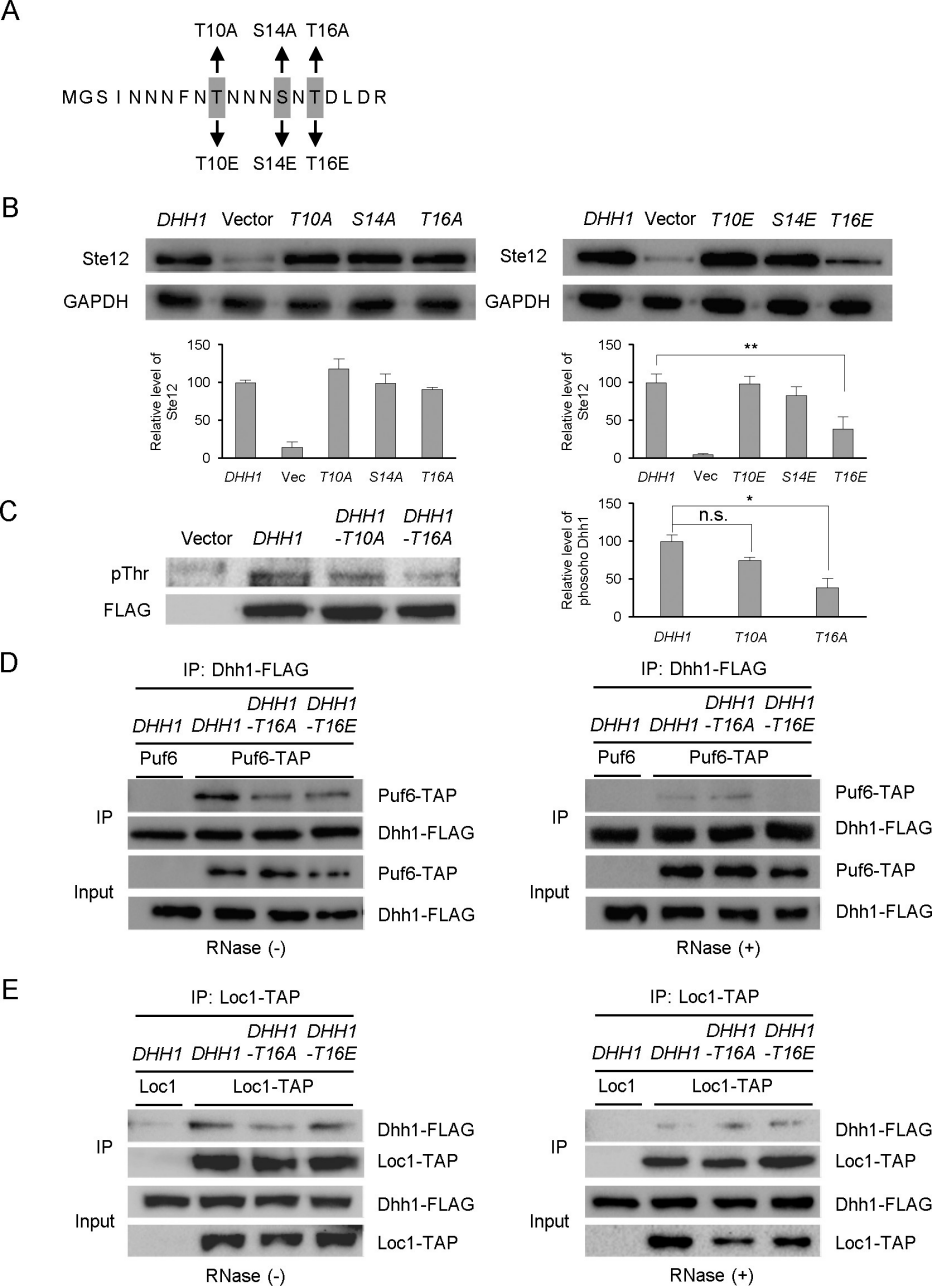
Effects of phosphorylation mutations of Dhh1 on Ste12 translation and Dhh1-Puf6 interactions. (A) Six mutations were introduced at the putative phosphorylation sites in the N-terminus of Dhh1. Mutated amino acids are indicated by gray boxes. (B) Ste12-HA protein levels in cells expressing various phosphorylation-mutant versions of Dhh1, as revealed by Western blot analysis. Protein extracts were prepared from the *dhh1* deletion strain (JK400) carrying a *DHH1* plasmid (pJI323), vector (pRS316), or mutant plasmid (pJI324-329). GAPDH was detected as a loading control. Graphs represent quantification of Ste12-HA to GAPDH ratio (n = 3 independent replicates). Values are mean ± SD. **p < 0.01. (C) Dhh1 phosphorylation was analyzed using an anti-phosphothreonine antibody. A wild-type BY4741 strain was transformed with *DHH1-*FLAG (pJI323), vector (pRS316), *DHH1-T10A* (pJI324), or *DHH1-T16A* (JI326) plasmids. Cell lysates were immunoprecipitated with anti-FLAG-conjugated agarose and probed with an anti-phosphor-threonine antibody. To detect Dhh1-FLAG, the membrane was re-probed with an anti-FLAG antibody. Graphs represent quantification of phosphorylated Dhh1 to Dhh1-FLAG ratio (n = 3 independent replicates). Values are mean ± SD. *p < 0.05. (D) Dhh1-Puf6 protein interactions, as revealed by co-immunoprecipitation analysis. Wild-type and *PUF6-TAP* were transformed with *DHH1*-FLAG (pJI330), *DHH1-T16A* (pJI331), or *DHH1-T16E* (pJI332). Cell lysates were immunoprecipitated with anti-FLAG-conjugated agarose in the absence (-) or presence (+) of RNase, and further probed with anti-PAP or anti-FLAG antibodies, respectively. (E) Dhh1-Loc1 protein interactions, as revealed by co-immunoprecipitation analysis. Wild-type and *LOC1-TAP* were transformed with *DHH1*-FLAG (pJI330), *DHH1-T16A* (pJI331), or *DHH1-T16E* (pJI332). Cell lysates were immunoprecipitated with anti-PAP-conjugated agarose in the absence (-) or presence (+) of RNase, and further probed with anti-PAP or anti-FLAG antibodies, respectively.

Phosphoproteomic analysis previously identified three phosphorylation sites of Dhh1: Thr10, Ser14 and Thr16 [29–31]. To determine whether the phosphorylation of these amino acid residues are crucial for the functions of Dhh1, we introduced either phospho-deficient (replacement with an alanine) or phospho-mimetic (replacement with a glutamate) mutations at Thr10, Ser14, and Thr16 (Fig 5A). We then examined whether these alterations of each Dhh1 phosphorylation site affected the expression level of Ste12. Phospho-deficient mutants at Thr10, Ser14, and Thr16 by alanine (T10A, S14A, and T16A) did not exhibit substantial defects in Ste12 expression, but the substitution of Thr16 by phospho-mimetic glutamate (T16E) dramatically decreased Ste12 expression (Fig 5B).

To directly analyze the in vivo phosphorylation status of Dhh1, we expressed wild-type and phospho-deficient (T10A and T16A) Dhh1-FLAG, collected these proteins using immunoprecipitation, and performed Western blot analysis with an anti-phosphothreonine antibody. We detected phosphorylation signals in the wild-type *DHH1* and *DHH1-*T10A cells, but this signal was significantly decreased in *DHH1-T16A* cells (Fig 5C). These results suggest that Dhh1 is phosphorylated in vivo, potentially at Thr16.

One possible mechanism through which Dhh1 phosphorylation may affect Ste12 expression is by altering a protein-protein interaction between Dhh1 and Puf6. To analyze whether there is a direct association between Puf6 and Dhh1, we generated cells carrying chromosomal TAP-tagged *PUF6* and plasmid-carried *DHH1-FLAG*, and compared the abilities of wild-type *DHH1*, *DHH1-T16A*, and *DHH1-T16E* to co-immunoprecipitate with Puf6 (Fig 5D). We observed that there was a protein-protein interaction between Dhh1 and Puf6, and that this interaction was decreased significantly by the *DHH1-T16E* mutation. When yeast extracts were treated with RNase, Dhh1 was not able to co-precipitate with Puf6, indicating that association of the two proteins was RNA-dependent. A protein interaction between Dhh1 and Loc1 was also observed, but it was not altered by the *DHH1-T16E* mutation (Fig 5E). These results demonstrate that the phosphorylation of Dhh1 at Thr16 may affect the Dhh1-Puf6 interaction.

### Phosphorylation site mutations of *DHH1* affect P-body accumulation

**Fig 6:**
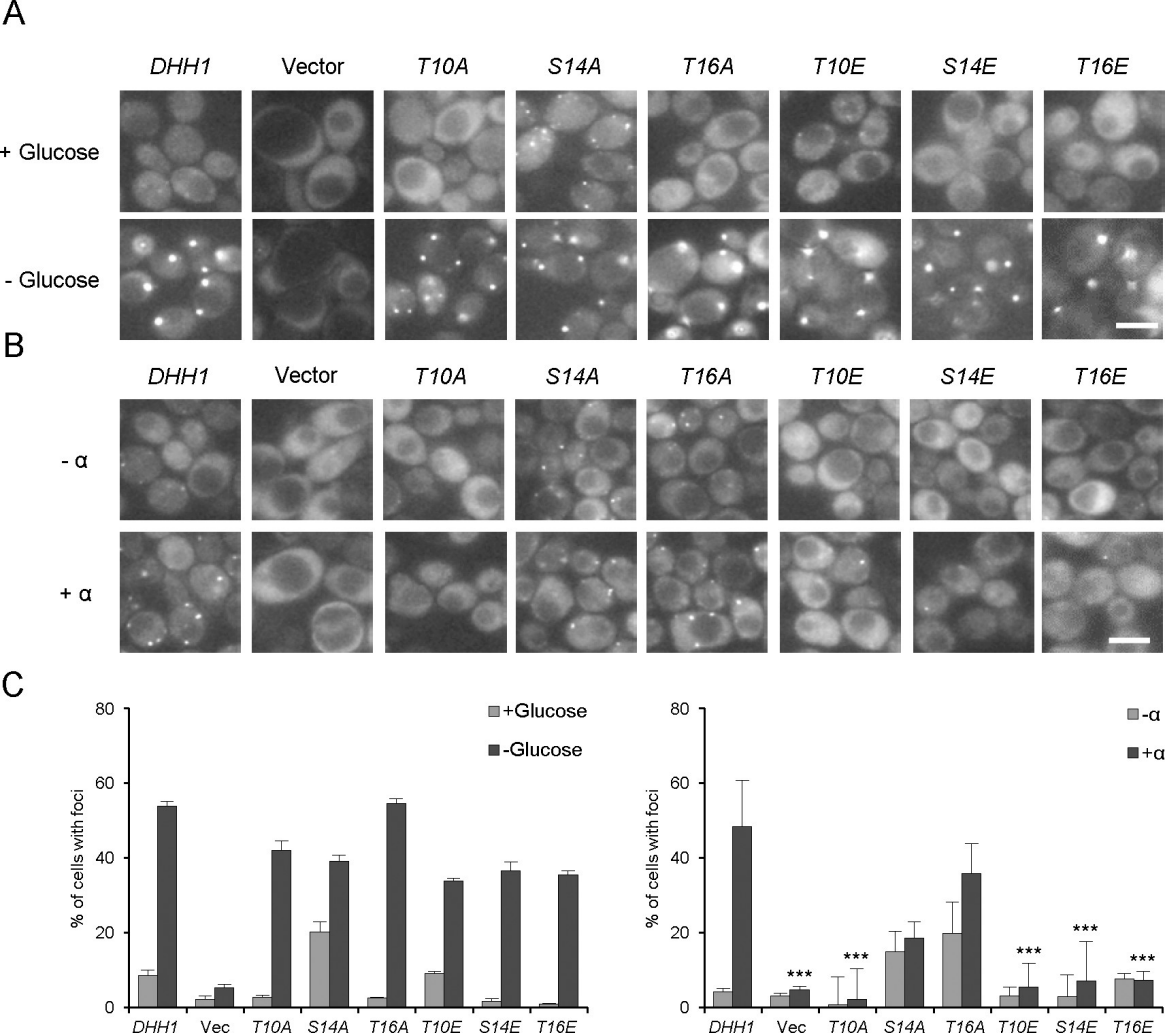
P-body accumulation in the Dhh1 phosphorylation mutants upon glucose deprivation and α-factor treatment. The *dhh1* deletion mutant strain (JK400) carrying DCP2::GFP (pRP1316) was transformed with the DHH1-FLAG plasmid (pJI323), vector (pRS316), or the various mutant plasmids (pJI324-329). (A) Cultures were grown in the absence (- α) or presence (+ α) of α-factor. (B) Cultures were grown in the presence or absence of glucose. Observations were made under an Olympus BX51 fluorescence microscope using a 100 X objective, and images were processed using the MetaMorph Program software. (C) Graphs represent the quantification of P-body containing cells (%), as conducted in (A) and (B) (n = 2 replicates, > 200 cells). Values are mean ± SD. *** p < 0.005.

Dhh1 is localized throughout the cytoplasm and can be redistributed into discrete foci, called P-bodies, upon various stresses [22,32]. P-bodies contain translationally repressed mRNAs and modulate the storage, decay, and translation of these mRNAs. In such granules, Dhh1 interacts with mRNA decapping enzymes (Dcp1/Dcp2), Xrn1 exoribonuclease, and decapping activators (e.g., Edc3, Scd6, and Pat1) [33,34]. The *dhh1* deletion mutation reportedly causes failure of P-body accumulation under glucose depletion or pheromone treatment [18,34]. To investigate whether Dhh1 phosphorylation alters the aggregation of P-body components, we used Dcp2-GFP as a representative P-body marker and examined P-body accumulation in our phosphorylation-site mutant strains (Fig 6). When treated with α-factor, the wild-type cells showed an increase in the number of P-bodies. Interestingly, the phospho-mimetic mutations (T10E, S14E, and T16E) and the phospho-deficient mutation, T10A, were associated with defects in P-body formation.The phospho-deficient mutant cells (S14A and T16A) showed increased levels of P-body accumulation under logarithmic-growth, compared with the wild-type cells. The effects of the phosphorylation site mutations of *DHH1* appear to be stress-specific, because the phosphorylation site mutants did not show any significant change in P-body accumulation under glucose deprivation (Fig 6B). These results suggest that the phosphorylation of Dhh1 affects its functions in P-body formation during α-factor treatment.

## Discussion

In this report, we show that Puf6 and Loc1 mediate the translational repression of the *STE12* mRNA, and that these roles are functionally connected with Dhh1, which acts as a positive regulator for *STE12* mRNA translation. Based on our results obtained from *dhh1loc1* and *dhh1puf6* double mutant strains and protein-protein interaction assays involving Dhh1, Loc1, and Puf6, we propose that the Puf6-Loc1 mediated repression of the *STE12* mRNA precedes the involvement of Dhh1 in this process (Fig 2D). Moreover, the phosphorylation of Dhh1 at Thr16 appears to be important for the physical and functional interactions of Dhh1 with Puf6 and Loc1.

The identification of three UUGU sequences (putative Puf6-binding target) upstream of the *STE12* start codon and the mutational analysis of these sequences support our contention that Puf6 and Loc1 regulate the translation of the *STE12* mRNA via its 5’-UTR. Puf6 and Loc1 bind directly the 3-’UTR of the *ASH1* mRNA in the presence of the She2 protein, and the formed complex is important for the bud-localization and translational control of this transcript [5,7]. The *STE12* mRNA has not been reported as a bud-localized transcript, and the Ste12 transcription factor is primarily localized in the nucleus [35,36]. Therefore, it seems likely that the mechanisms underlying Puf6- or Loc1-dependent repression differ depending on the target mRNA(s) [7,37]. In addition, the regulatory factors that associate with Puf6 or Loc1 in the target mRNA-protein complexes are likely to affect this repression mechanism.

A recent report showed that Puf6 and Dhh1 act together in the translational repression of *ASH1* mRNA with Dhh1 binding to the 5’-UTR of the *ASH1* mRNA and interacting with Puf6 [38]. Here, we show that Dhh1 and Puf6 interact on both genetic and protein levels to mediate the translation of the *STE12* mRNA. In particular, our phospho-mimetic substitution of the phosphorylation site residue, Thr16, in the N-terminus of Dhh1 (*DHH1*-T16E) dramatically decreased Ste12 expression and the Dhh1-Puf6 protein interaction. We observed that this Dhh1-Puf6 interaction appeared to be RNA-dependent. We hypothesize that the binding of Puf6 to the 5’-UTR of the *STE12* mRNA suppresses its translation. The interaction of Dhh1 with mRNA-bound Puf6 may be critical for the release of the Puf6-mediated repression.

Dhh1 directly interacts with both decapping and deadenylase complexes [22, 34]. Our observation that the phosphorylation site mutations of *DHH1* showed defects in P-body accumulation upon mating factor treatment but not under glucose deprivation prompted us to further question whether the N-terminal phosphorylation regulates the interaction of Dhh1 with P-body components, such as the decapping and deadenylase complexes.

The Dhh1 N-terminal region was not included in previous crystal structure or functional domain analyses of Dhh1, and we know little about the roles of the putative phosphorylation sites in this region [20,30,31,39,40]. Our present observation that the N-terminal phosphorylation sites of Dhh1 regulate its association with Puf6 might improve our understanding of the repression mechanisms that are mediated by Puf6, Loc1, and Dhh1.

## Materials and methods

### Strains and growth conditions

The *Saccharomyces cerevisiae* strains and plasmids used in this study are listed in Table 1. Double-deletion mutants JK460 and JK461 were derived from JK443 and JK444, respectively, using a PCR-based gene disruption method. Briefly, the *dhh1* disruption cassette containing the *LEU2* marker was amplified, and the obtained PCR products were transformed into the *puf6* or *loc1* deletion strains [18].

**Table 1:** Strains and plasmids used in this study.

Strains	Genotype	Reference
JK147	*MAT a ura3-52 leu2-3*, *112 trp1-1*, *ade2*, *cyh* ^*r*^	[18]
JK400	*MAT a dhh1*::*LEU2 ura3-52 leu2-3*, *112 trp1-1*, *ade2*, *cyh*^*r*^	[18]
BY4741	*MAT a his3Δ1 leu2Δ0 met15Δ0 ura3Δ0*	Euroscarf^a^
JK433	*MAT a his3Δ1 leu2Δ0 lys2Δ0 ura3Δ0 dhh1*::*kanMX4*	Euroscarf
JK443	*MAT a his3Δ1 leu2Δ0 lys2Δ0 ura3Δ0 loc1*::*kanMX4*	Euroscarf
JK444	*MAT a his3Δ1 leu2Δ0 lys2Δ0 ura3Δ0 puf6*::*kanMX4*	Euroscarf
JK460	*MAT a ura3Δ0 leu2Δ0 his3Δ1 met15Δ0 ura3Δ0 loc1*::*kanMX4 dhh1*::*LEU2*	This work
JK461	*MAT a ura3Δ0 leu2Δ0 his3Δ1 met15Δ0 ura3Δ0 puf6*::*kanMX4 dhh1*::*LEU2*	This work
JK445	*MAT a his3Δ1 leu2Δ0 lys2Δ0 ura3Δ0 LOC1*::*TAP*	Open Biosystems^b^
JK446	*MAT a his3Δ1 leu2Δ0 lys2Δ0 ura3Δ0 PUF6*::*TAP*	Open Biosystems
Plasmid	Genotype	Reference
pJI255	*STE12*::*HA URA3 CEN*	[19]
pJI256	*CLN1*::*HA URA3 CEN*	[19]
pJI275	*pGAL-STE12*::*HA URA3 CEN*	This laboratory
pJI277	*DHH1 URA3 CEN*	[19]
pJI323	*DHH1*::*FLAG URA3 CEN*	This work
pJI324	*DHH1-T10A*::*FLAG URA3 CEN*	This work
pJI325	*DHH1-S14A*::*FLAG URA3 CEN*	This work
pJI326	*DHH1-T16A*::*FLAG URA3 CEN*	This work
pJI327	*DHH1-T10E*::*FLAG URA3 CEN*	This work
pJI328	*DHH1-S14E*::*FLAG URA3 CEN*	This work
pJI329	*DHH1-T16E*::*FLAG URA3 CEN*	This work
pJI330	*DHH1*::*FLAG URA3 2μ*	This work
pJI331	*DHH1-T16A*::*FLAG URA3 2μ*	This work
pJI332	*DHH1-T16E*::*FLAG URA3 2μ*	This work
pJI333	*STE12*::*HA TRP1 CEN*	[18]
pJI352	*GPA2*::*HA URA3 CEN*	This work
pJI354	*CLN1*::*HA TRP1 CEN*	This work
pJI355	*GPA2*::*HA TRP1 CEN*	This work
pJI356	*LOC1 LEU2 CEN*	This work
pJI357	*PUF6 LEU2 CEN*	This work
pJI377	*UACU*^*124*^ *-STE12*::*HA URA3 CEN*	This work
pRS426	*URA3 2μ*	[41]
pRS314	*TRP1 CEN*	[42]
pRS316	*URA3 CEN*	[42]
pRP1316	*DCP2*::*GFP TRP1 CEN*	[43]

^a^ Euroscarf Collection Center (Frankfurt, Germany)

^b^ Open Biosystems (Colorado, USA)

Yeast strains were grown in YEPD (1% yeast extract, 2% peptone, 2% glucose) or SC (synthetic complete; 0.67% yeast nitrogen base w/o amino acid, 2% glucose, all required amino acids) medium. For galactose induction, cells were grown in SC medium containing 2% raffinose to the early log phase (OD_600_ = 1.0) and induced with 2% galactose or 2% galactose plus 0.2% glucose for 2 hrs. For α-factor induction, cells were grown in SC medium to the early log phase (OD_600_ = 0.5), and treated with α-factor (5 μM, 1:100 dilution of a 0.5 mM solution in methanol; Sigma-Aldrich, St. Louis, MO, USA) for 30–90 min. For glucose deprivation, cells were centrifuged and washed with SC lacking glucose. Pellets were resuspended in SC lacking glucose and incubated for 10 min.

### Plasmid construction and site-directed mutagenesis

The *DHH1*-FLAG plasmid (pJI323) was constructed using overlap-extension PCR method. Two PCR-amplified products sharing the FLAG sequence were assembled by PCR and subsequently cloned into the pRS316 vector. Phosphorylation site mutations for Dhh1 were generated in pJI323 by using mutagenic oligonucleotide primers (Table 2). After PCR amplification, the template DNA was digested with D*pn*I. All mutant constructs were confirmed by sequence analysis. pJI377 plasmid was constructed by using mutagenic oligonucleotide primers and pJI255 template.

**Table 2:** Primers used in this study.

Primer name	Sequence
Dhh1-F	GGGCTGCAGGACCAACAAACCAATA
Dhh1-R	GGGACTAGTGTGATAGTAATAAAA
Dhh1-Flag-F2	GTATCGATGGATTACAAGGATGACGACGATAAGATCTAAAGAATATCTAAGAAA
Dhh1-Flag-R2	GATCTTATCGTCGTCATCCTTGTAATCCATCGATACATACTGGGGTTGTGACTG
Dhh1-T10A F	CCATCAATAATAACTTCAACGCTAATAATAACAGTAATACGG
Dhh1-T10A R	CCGTATTACTGTTATTATTAGCGTTGAAGTTATTATTGATGG
Dhh1-S14A F	CTTCAACACTAATAATAACGCTAATACGGATCTCGATCGG
Dhh1-S14A R	CCGATCGAGATCCGTATTAGCGTTATTATTAGTGTTGAAG
Dhh1-T16A F	CTAATAATAACAGTAATGCGGATCTCGATCGGGAC
Dhh1-T16A R	GTCCCGATCGAGATCCGCATTACTGTTATTATTAG
Dhh1-T10E F	CCATCAATAATAACTTCAACGAAAATAATAACAGTAATACGG
Dhh1-T10E R	CCGTATTACTGTTATTATTTTCGTTGAAGTTATTATTGATGG
Dhh1-S14E F	CTTCAACACTAATAATAACGAAAATACGGATCTCGATCGGG
Dhh1-S14E R	CCCGATCGAGATCCGTATTTTCGTTATTATTAGTGTTGAAG
Dhh1-T16E F	CTAATAATAACAGTAATGAGGATCTCGATCGGGAC
Dhh1-T16E R	GTCCCGATCGAGATCCTCATTACTGTTATTATTAG
UACU^124^-Ste12 F	CAGGTAAGCACTGAAGACTACTTTTATAAGTGTCCCAAGCG
UACU^124^-Ste12 R	CGCTTGGGACACTTATAAAAGTAGTCTTCAGTGCTTACCTG
STE12-qRT F	GTATCTCCTAGCGACCCTAC
STE12-qRT R	AGTTTGCTGGCCAGAGTTGT
ACT1-qRT F	CTGCCGGTATTGACCAAACT
ACT1-qRT R	CGGACATAACGATGTTACCG
ASH1-RIP F	ACGACGCTTAGAGGAGTAGA
ASH1-RIP R	GTTGTCGAGTTTCATCACCA
STE12-RIP F	CAGTAAAGCTACTCCGGGCGA
STE12-RIP R	AGGCGGGCTCATTAACGGGC

### Western blot analysis

Total protein preparation and Western blotting were performed as previously described [18]. HA-tagged proteins were detected with an anti-HA monoclonal antibody 12CA5 (Roche, Manheim, Germany). Horseradish peroxidase-conjugated anti-mouse antibody (Santa Cruz Biotech, Santa Cruz, CA, USA) was utilized as the secondary antibody. Tubulin protein was detected using a monoclonal anti-α-tubulin antibody (Sigma-Aldrich). Protein bands were detected using the EPD^TM^ Western reagent (Elpis-Biotech, Daejeon, KR).

### Total RNA extraction and qRT-PCR

Total RNA was prepared as described previously [18]. cDNA was synthesized from total RNA by reverse transcriptase (RT) using an M-MLV cDNA synthesis kit (Enzynomics, Daejeon, Kr). *STE12* mRNA levels were measured by quantitative real-time PCR (qRT-PCR) using SYBR green I (GenetBio, Daejeon, Kr). All values were normalized to the level of *ACT1* (actin) mRNA. *STE12-*specific primers and *ACT1*-specific primers (Table 2) were used to amplify 100-bp fragments of each coding sequence.

### Fluorescence microscopy

Cells were treated with α-factor or grown under glucose-deprived conditions and observed under an Olympus BX51 Fluorescence Microscope using a 100 X objective. The images were processed using the MetaMorph Program software.

### Immunoprecipitation and phosphorylation assay

Cells were grown in SC medium to logarithmic phase (OD_600_ = 1.0), harvested, and lysed with glass beads in lysis buffer (50 mM HEPES/KOH, pH 7.5, 140 mM NaCl, 1 mM EDTA, 1% Triton X-100, 1 mM PMSF, 10 mM NaF, 1 mM Na_3_VO_4_). Immunoprecipitation was carried out using an anti-Flag antibody (Sigma-Aldrich, St. Louis, MO, USA). Briefly, 20 μl of protein A/G beads (Santa Cruz Biotech) were added to 1 ml of freshly prepared lysate and incubated for 2 hours on a turning wheel at 4°C. Immunopellets were washed seven times in lysis buffer and recovered by boiling in 2X SDS buffer for 10 min at 100°C. For co-immunoprecipitation experiments, Dhh1 was detected using a monoclonal anti-FLAG antibody (Sigma-Aldrich). Puf6 and Loc1 were detected using a monoclonal anti-PAP antibody (Sigma-Aldrich, St. Louis, MO, USA). HRP-conjugated anti-mouse antibody (Santa Cruz Biotech) was utilized as a secondary antibody.

### RNA immunoprecipitation (RIP)

Cells were grown to logarithmic phase (OD_600_ = 1.0), harvested, and lysed with glass beads in 200 μl FA lysis buffer (50 mM HEPES/KOH, pH 7.5, 140 mM NaCl, 1 mM EDTA, 1% Triton X-100, 0.1% Sodium deoxycholate, 0.4 U/μl RNasin, 1X protease inhibitor cocktail). Immunoprecipitation was carried out using an anti-PAP antibody (Sigma-Aldrich, St. Louis, MO, USA) and protein G-sepharose bead (GE healthcare, Chicago, IL, USA). Immunopellets were washed three times and suspended in ChIP elution buffer (100 mM Tris-Cl, pH 8.0, 10 mM EDTA, 1% SDS, 0.4 U/μl RNasin). The 5M NaCl and 0.1 μg/μl proteinase were added to 150μl of supernatant and the mixture was incubated at 42°C for 1 hr, and then at 65°C for 1 hr. RNA preparation was carried out as described above and RT-PCR were carried out using *STE12-*specific or *ASH1*-specific primers (Table 2).
